# Exploring the Potential for Use of Virtual Reality Technology in the Treatment of Severe Mental Illness Among Adults in Mid-Norway: Collaborative Research Between Clinicians and Researchers

**DOI:** 10.2196/13633

**Published:** 2019-06-10

**Authors:** Solveig Osborg Ose, Hilde Færevik, Jannike Kaasbøll, Martin Lindgren, Kristin Thaulow, Stig Antonsen, Olav Burkeland

**Affiliations:** 1 SINTEF AS Digital Trondheim Norway; 2 Coperio AS Trondheim Norway; 3 Trondheim Municipality Trondheim Norway; 4 St. Olavs University Hospital Trondheim Norway

**Keywords:** virtual reality, severe mental illness, collaborative research, technology, social work

## Abstract

**Background:**

Virtual reality (VR) technology is not currently used in the treatment of severe mental health illness in Norway.

**Objective:**

We aimed to explore the potential of VR as a treatment for severe mental health illness in Norway, through collaborative research between clinicians and researchers.

**Methods:**

A collaborative research team was established, comprising researchers, the manager at a district psychiatric center, and the manager of the local municipal mental health service. An all-day workshop with eight clinicians—four from specialist mental health services and four from municipal mental health services—was conducted. The clinicians watched three different VR movies and after each one, they answered predefined questions designed to reflect their immediate thoughts about VR’s potential use in clinical practice. At the end of the workshop, two focus group interviews, each with four clinicians from each service level, were conducted.

**Results:**

VR technology in specialist services might be a new tool for the treatment of severe mental health illness. In municipal mental health services, VR might particularly be useful in systematic social training that would otherwise take a very long time to complete.

**Conclusions:**

We found substantial potential for the use of VR in the treatment of severe mental health illness in specialist and municipal mental health services. One of the uses of VR technology with the greatest potential was helping individuals who had isolated themselves and needed training in social skills and everyday activity to enable them to have more active social lives. VR could also be used to simulate severe mental illness to provide a better understanding of how the person with severe mental illness experiences their situation.

## Introduction

A current challenge in the development of technology for mental health is that it is largely market-driven. This means that large target groups, such as people with less severe anxiety and depression disorders who are willing and able to pay, receive ready access to new supporting technology. People with severe mental disorders, lower levels of functioning, and poorer ability to pay often are not involved in commercial technology development; therefore, they receive little benefit from these technological advances.

In Norway, less severe, common mental illnesses must be regarded as public diseases because they have such a high incidence [1]. The sheer volume of these conditions indicates that treatment that can be delivered using commonly available technology such as smartphone apps will be a sensible option. More than 10,000 mental health–related apps are available for download. Because very few of these are evidence based, more transparency and trust through better oversight and a stronger commitment to research are needed [2]. Although some argue that greater patient and clinician involvement is needed to evaluate digital technologies and ensure they target unmet needs, maintain public trust, and improve clinical outcomes [3], the results of treatments using such technology have already shown some promise. A meta-analysis showed that psychological interventions delivered via smartphone devices can reduce anxiety [4], and a recent randomized controlled trial concluded that users of the mental health apps MoodMission and MoodKit experienced decreases in depression [5]. A survey aimed at exploring mental health stakeholders’ knowledge, acceptance, and expectations of digital treatments for depression found that digital treatments were rated as more acceptable for milder forms of depression [6].

The use of technology in the treatment of severe mental disorders is less developed and has received less attention. One of the reasons for this may be the lack of interest from commercial developers because the market is smaller and those who might benefit have a lower ability to pay. Persons with serious mental illness are found to earn, on an average, one-third less than the median earnings, with no significant between-country differences [7].

Most digital treatments are forms of cognitive behavior therapy, that is, they are essentially existing treatments that have been converted into digital interventions. Truly novel digital treatments are infrequent [8]. Examples of novel digital treatments, according to Fairburn and Patel, include virtual reality (VR)–based treatments that are found to be effective for individuals with a range of severe mental health problems [9]. Valmaggia at King’s College found that the innovative potential of VR is that it allows the measurement of real‐time cognitive, emotional, physiological, and behavioral responses to a variety of real‐life situations while enabling experimental control [10]. Preliminary findings suggest that VR can be applied to the delivery of cognitive rehabilitation, social skills training interventions, and VR-assisted therapies for psychosis [11]. A pilot validation study tested a new VR social situation paradigm (a party in a bar) with a subsample of participants who scored high or low in trait paranoia and found that the VR scenario could be used as a psychological assessment and treatment tool for people who experience paranoia in social situations [12]. Enthusiasm is growing among clinicians and researchers worldwide about the potential that VR offers for improving the assessment and treatment of mental and physical health problems [10].

However, while some clinicians explore the possibility of using new technology in the treatment of severe mental illness with good results, it may be difficult for other clinicians to introduce the technology into their own practice. If the barriers to the use of technology for behavioral health care, such as the characteristics of the technology, potential end users, organization structure and climate, and factors external to organizations [13], are to be overcome, a collaborate research methodology including managers and clinicians in mental health services might be warranted. Long-term collaborative research between researchers, managers, and clinicians was established with the aim of taking the first steps in exploring the potential for the use of VR technology in the treatment of severe mental illness among adults in Norway. Finance for a start-up project was provided by the Regional Research Fund for mid-Norway.

## Methods

Collaborative research projects have emerged as a particular form of academia–industry interaction [14]. Our research plan included the following steps: (1) establish trust and robust personal relationships between main stakeholders, (2) obtain financing for a preproject, and (3) include clinicians to explore the potential for using VR in mental health services. If potential was found in step 3, we planned to establish a main project based on the same collaboration.

The collaboration began with several informal meetings between the researchers and the two management representatives from the district psychiatric center (DPC) and the municipal mental health care service. Three researchers were involved at the beginning: a social scientist with extensive mental health services research experience; a senior scientist who specializes in sensor technology, wearables, and physiology; and a scientist with a PhD in clinical medicine in mental health. The researchers also met with some of the clinicians at the DPC and discussed their thoughts and plans to establish a collaborative research group on VR in mental health treatment. A senior scientist who is a technical VR and augmented reality expert was involved from the beginning and will be further involved if the first step of the project shows potential among clinicians and managers.

The group was further expanded with two clinical psychologists experienced in creating and testing VR content in the commercial market outside health services. Their experience included working with the police (mental training using VR), a private bank (emotional intelligence training using VR), municipalities, and schools (using VR scenario training to assess risk in relation to violence) and biofeedback training in VR in the pain clinic of a university hospital. They were employed by a private local company called Coperio that provided management counselling services, innovation, occupational health services, and outpatient mental health treatment for less severe problems.

A workshop with clinicians was planned to explore the potential of VR technology in the treatment of severe mental illness in adults in Norway. The 6-hour workshop was organized by the researchers and two clinical psychologists.

Eight clinicians, four from the DPC and four from the local mental health service, attended the workshop (seven men and one woman). The participants were recruited by the managers at the DPC and the local mental health service. Five of the participants had some experience in the use of VR privately, while three did not. All eight participants signed a written consent form.

The workshop started with an introduction to the research in the field, especially the research from the United Kingdom and Valmaggia. The local company then shared some of their experiences with the use of VR in the commercial market (ie, customers willing to pay for the use of VR in training and personal development, such as the private banking market). They also introduced the eight participants to the equipment and showed them how it worked. After a coffee break, we divided the participants into two groups and placed them in separate rooms. We showed them Movie 1 and then asked them to write down their immediate thoughts. After lunch, the participants watched Movies 2 and 3 and were asked to write down their immediate thoughts after each one. At the end of the day, the researchers conducted focus group interviews with both groups.

The study design was preapproved by the Norwegian Centre for Research Data (project number: 845033). The interviews were recorded and later transcribed. Thereafter, the research team members met to discuss the experiences of the day and plan further collaborations.

In this initial set-up, we used simple and affordable mobile VR equipment (Samsung Gear VR Oculus, Menlo Park, CA) provided by the local company.

The three short example movies were of (1) an angry man at the office, (2) a self-presentation to a small group in a work setting, and (3) mindfulness on the beach with biofeedback.

The first film was a VR scenario filmed by Coperio about an angry man coming into the participant’s office. He gradually becomes more threatening and knocks his fist on the table. The film lasts 1 minute and 36 seconds and does not include biofeedback.

The second VR film was more advanced and included biofeedback. The film shows four people sitting at a round table in an office meeting setting. A man and a woman first present themselves in a relatively formal matter, following which the VR user presents himself/herself. A wristband measures and records the VR user’s pulse as a measure of heart rate throughout the scenario. After the film, the VR users can see how their pulse rate developed during the session and thus train to reduce their nervousness in such situations. The VR users also received questions triggering self-reflection, including what they recalled about the content of the other meeting participants’ presentations.

The third film showed an animated tropical beach with a bird on the water. When the viewer’s pulse is calm, the waves will be calm and the bird will approach. When the viewer’s pulse increases, the number of waves increases, the bird floats away from the shore, and the wind and sound increase [15].

The short questionnaire included one question to be answered before the participants watched any of the movies: “What are your expectations about the use of VR in clinical practice?” After each film, the participants answered the following questions: (1) “What are your immediate thoughts after watching the scenario?” (2) “What could be better?” (3) “What do you think was good?” and (4) “What are your thoughts about the potential use and application of VR in the treatment of severe mental illness?” At the end of the testing, they also answered the question, “Have you changed your mind about the use of VR in mental health care during the day?”

## Results

In the following subsections, we combine the information given in the short questionnaires and the focus group interviews.

### Expectations of Virtual Reality Before the Workshop

In general, the participants reported that they had mixed expectations of VR in mental health treatment. One participant argued that the technology has potential for creating and adjusting scenarios that may be important in patient training and that allow repetition in a safe environment. Another said that VR could be part of future mental health services and a new tool available to clinicians. A third participant thought that VR had a place in mental health treatment, but that it would take 10-20 years before this potential would be realized. Others thought that VR had potential in many different settings, including social training or learning to understand emotional expressions among persons with severe mental illness. Another participant made the point that it would be easier to get training started in VR because exposure to the real word is very hard for many of those with severe mental illness. Several participants mentioned that training in a safe environment had the potential to produce larger treatment effects.

### Experiences with the Films

The films were used as examples of the types of films that could be developed and as a means of triggering the respondent’s creativity in their thinking of ways that VR could be used to improve the lives of persons with severe mental illness.

#### Scenario 1: An Angry Man at the Office

The participants found the film realistic. Some of the participants thought the situation was uncomfortable and felt immersed in the situation. Others did not feel immersed. There were comments about the need to improve graphic and sound quality, which may increase immersion. Suggested areas of application included training employees in public services including mental health services, training in conflict management, and training in handling other peoples’ extreme emotional reactions. Another suggestion was that such films could be used in the treatment of anger disorder, where patients can see and feel how they affect other people with their anger behavior. It was suggested that situations that trigger the emotion of anger could be filmed, so that the patients could practice on specific problems and personal triggers as a form of personalized treatment.

#### Scenario 2: Self-Presentation

The participants also found this film realistic and thought the scenario was an example of a very common situation. In the film, one of the people in the meeting arrives late and one of the participants found himself to be slightly irritated by that person. Another commented that the feeling of going to a meeting without knowing the agenda was not common. All the participants found it interesting to see how their heart rate developed throughout the meeting, and for most of them, the pulse rate increased when it was their turn to present themselves. One of the clinicians reflected on the fact that his attention shifted toward himself and that he did not pay much attention to the others in the meeting. The participants all agreed that this was a realistic scenario that everybody could learn something from.

Suggested applications included training in similar situations to improve users’ ways of presenting themselves and to learn to pay more attention to what other people communicate. It was also suggested that this could be used in couples therapy, the treatment of social phobia, and general training to reduce performance anxiety.

#### Scenario 3: Mindfulness on the Beach With Biofeedback

One of the participants thought this exercise was somewhat disturbing, as the bird floating in the water was big and spooky. This participant also felt that there was something behind the chair he was sitting on at the beach. One clinician suggested that the scenario could be used as part of the treatment for attention-deficit hyperactivity disorder and posttraumatic stress disorder (PTSD), because these patients often have a high pulse rate without being aware of it, and this mindfulness exercise could be a way of learning more about their own reactions. Another clinician thought it was funny that the image could be altered by thinking about situations that increased the pulse rate, while another found the exercise relaxing. Some said it would be better if the picture was real rather than animated and that the quality of the graphics and sound needed improvement. One observation was that the scenario focused on punishment rather than reward, as patients were punished with more waves and darkness if they could not control their pulse. All participants were fascinated by the interaction between biofeedback and the VR film.

Suggested applications included training to focus on the pulse and breathing and learning how to relax (mindfulness), autogenic training, and stabilization of overactivation in patients with PTSD. One of the clinicians suggested that this could be helpful for those with severe anxiety disorder:

Learning to control the heart rate can be useful for many patients who complain of strong anxiety and are very uneasy and afraid all the time. Perhaps the experience that it is possible to feel that one is becoming calm is useful.

#### After Testing Virtual Reality

Six people were more positive about VR in mental health care after testing VR themselves, while two people who were initially positive had not changed their view during the day. All participants concluded that they could see opportunities for using VR in the treatment of mental health illness, especially in skill training and providing safe exposure to different situations. One of the participants said:

I am positive about using VR in mental health care. The day today has given me a glimpse into the opportunities this technology holds. This exciting technology should be developed further and applied to user groups that can make use of it.

### General Findings

In the interviews, we asked the participants about the general service attitude toward the use of VR in mental health care. Most of them observed a certain expectant attitude among their colleagues; for example, some cases presented about VR in the media (typically outside the health services), which may have contributed to a more positive attitude toward the technology. Few of the clinicians thought that the patients would be skeptical to try VR in treatment:

I do not think the patients in the future will be sceptical towards VR. And I also think that because the youth today get an iPhone almost at birth, VR may be an incredible opportunity in the treatment of future patients.

At the beginning of the day, the participants had watched a film we received from Valmaggia, showing how VR technology was used in clinical practice at King’s College. Several of the clinicians had started to think about the added value of using VR in clinical practice:

I see something valuable with this method, that you can sequentially put people into an actual situation. When I tried [VR today], I got a very strong experience of being in the situation, it was very realistic to me. And you can take the patient out of the situation again without stress to reflect. I think that the Valmaggia method is fantastic.

They also added that being able to go back and see the same sequence and try to observe things that were not observed the first time was valuable.

One participant with extensive experience in mental health care said that about 20 years ago, he worked with persons with severe mental illness, training them on social skills such as how to keep a friendship, end a poor relationship, or a conversation:

Such social skill training was very demanding. Difficult with the setting, because how should we communicate to get people to contribute to this? It would have been much easier if you could use the VR technology.

The clinicians also mentioned the need for social training for a large group of patients with different forms of avoidant personality disorder and provided examples of functionalities that they could build into a VR setting:

Small talk and situations of everyday life, such as taking the bus. Buying the bus ticket. Ordering a cup of coffee. Not very complicated things, but rather [things to] try to build up their social skills.

It would be really good if someone got some practice in expressing their needs or disagreeing with some things. Yes, as training in taking up some space, and being conscious about what kind of space you take.

We also discussed the situation for many people with severe mental illness who receive only acute treatment in specialist mental health services, because there is no other effective treatment available. These people often isolate themselves in their homes and have a poor social life. The effect of using VR to prevent isolation was suggested:

And being able to get out of the door, pick up the newspaper, nod to the neighbour and then go the next step, to make contact with someone on the sidewalk or a neighbour, to build something that can be anti-isolating. Because we know the specialist health service must contribute when the psychosis worsens.

One of the participants working in the municipal mental health services added:

Training situations could be split into suitable sequences and we could simply achieve that they open the door when mental health carers were at the door.

One of the psychologists from the local company had previously worked in specialist mental health services and told a story where he guided a person with severe mental illness in skills training. At first, the patient could only manage to put his head out of the door and look down the hallway before closing the door. The goal for the patient was to read the paper in a local café. He eventually reached the goal, but the process took 2 years. The clinician estimated that if he had VR equipment available at the time, the goal could have been reached in two months. Another clinician had a similar case:

I think VR is a tool for improving the life of some people with much suffering. I had a person who was terrified of going on bridges and when you live in this city with many bridges, this fear makes your life quite difficult, so we practiced by walking on bridges. If we had VR, we could have achieved our goals much faster.

We also considered that many of those with severe mental illness lack the ability to read human signals correctly:

Misinterpretation of signals is quite widespread among persons suffering from severe mental illness. What they read as a rejection signal, do we read as others being a little questioning, perhaps? If the situation could be simulated and we had the ability to go back to reflect, what was it? Did he say he wouldn’t see me anymore? No, he didn’t.

We also discussed how VR might be used to train patients that had never been working or had been outside the labor market for many years, to begin or return to work. A library of film jobs could be made, and the person could train in VR to increase their readiness to start working. Many of those with severe mental illness are not in employment, and it was suggested that VR training could be introduced into the individual placement and support efforts of the labor and health services.

We also discussed the possibility of simulating severe mental illness in VR so that, for example, relatives and others could gain a better understanding of how it felt to have this illness and thus improve the situation for those with severe mental illness. One participant said:

I think this would be very useful. Relatives are the ones who want the most and they need and are able to understand and take a role, a constructive role in this. And this is anti-isolating thinking. I think this is a pretty good focus.

Another clinician added:

From being desperate and furious to desperate and furious all the time, parents could have a VR experience where they see what it looks like for their son or daughter. It would give a better understanding of the situation and perhaps be easier to comprehend.

## Discussion

### Principal Findings

There is still a long way to go to promote investment, resources, and accountability in the mental health sector. It is suggested that the next steps include enhanced international co-operation and the creation of private–public partnerships, specifically with technology companies [16]. It is also argued that advancements in Web, mobile, sensor, and informatics technologies can help us better understand the very nature of mental illness and revise our fundamental assumptions about the structure, boundaries, and modalities of mental health treatment [17].

Long-term collaborative research groups between health care management representatives, clinicians, technology partners, and researchers are one way to increase innovation, dissemination, and adaption of new treatment forms in mental health services. This prestudy is the small beginning of such a long-term collaborative research group. The first aim of the research group was to explore the potential for the use of VR technology in the treatment of severe mental illness in adults. We conclude that there is potential in both specialist mental health services and municipal services in Mid-Norway.

A main concern is that individuals living with severe mental illness are often difficult to engage in ongoing treatment and show high dropout rates and poorer clinical outcomes, with symptom relapse and rehospitalization [18]. VR technology might be a new tool for the treatment of severe mental illness in specialist mental health services. Current research on mental health and the use of VR is related to social anxiety disorder or social phobia [19-23], psychosis [11,24], and high-functioning autism [25-27] and intellectual disabilities in young adults [28]. We found only a few studies that explored the use of VR in social skills training for persons with severe mental illness [29-31]. However, isolation, belonging, and social cognition are old problems that, despite new medicine, we do not have satisfactory answers to. The opportunity to train in virtual scenarios without paying the social price in real-life situations can open the door to interesting studies.

Pure phobias such as arachnophobia or agoraphobia seem to be the easiest to cure using VR. However, many of the patients in mental health services have several diagnoses and a complex combination of different problems. In line with the thoughts of Kinderman (2014) and, in Norway, Aarre (2010), we found that modern mental health services must base their work on the fact that distress is usually an understandable reaction to life’s challenges and that diagnoses should be replaced by descriptions of the individual’s problems [32,33].

The research group has decided to continue the collaboration and has established a long-term regional collaboration to explore and test the use of VR, artificial intelligence, and other technologies in the treatment of severe mental illness. The region is known as Norway’s technological capital, with Scandinavia’s biggest research institution—SINTEF—located in Trondheim, Norway’s third-largest city. A research collaboration has also been established between SINTEF, King’s College, and Valmaggia.

### Conclusions

We conclude that there is potential for VR in the treatment of severe mental illness in our region. One of the largest opportunities this collaborative research group identified so far was the use of VR technology to help individuals who have isolated themselves and need training in social skills and everyday activity to enable them to have a more active social life. We also believe that VR could be used to simulate severe mental illness, so that staff, relatives, and others could get a better understanding of how persons with severe mental illness experience their situation.

## Abbreviations

VR: virtual reality
DPC: district psychiatric center
PTSD: posttraumatic stress disorder
